# INFLUENCE OF ASPIRIN ON AGING SKELETAL MUSCLE SIZE

**DOI:** 10.1093/geroni/igac059.2888

**Published:** 2022-12-20

**Authors:** William Fountain, Masatoshi Naruse, Alex Claiborne, Holmes Finch, Scott Trappe, Todd Trappe

**Affiliations:** Ball State University, Muncie, Indiana, United States; Ball State University, Muncie, Indiana, United States; Ball State University, Muncie, Indiana, United States; Ball State University, Muncie, Indiana, United States; Ball State University, Muncie, Indiana, United States; Ball State University, Muncie, Indiana, United States

## Abstract

Aspirin is one of the most commonly consumed cyclooxygenase (COX)-inhibitors and anti-inflammatory drugs and may provide insight into the control of age-related skeletal muscle atrophy. This investigation compared skeletal muscle size (via computed tomography) of older individuals from the Health ABC study that did not consume aspirin or any other COX-inhibiting drug (non-consumers; n=1,155, 74±3y, 48% women, 45% black) to those that consumed aspirin daily (and not any other COX-inhibiting drug) and for at least one year (aspirin consumers; n=515, 74±3y, 39% women, 30% black, average aspirin consumption: 6y). In white and black men, the long-term aspirin consumers had significantly larger quadriceps muscle size compared to non-consumers (p < 0.05). In white men only, these differences were influenced by age (p < 0.05). In white and black women, quadriceps muscle size was not different between the aspirin consumers and non-consumers (p>0.05). In men and women of either race, there was no hamstrings muscle size difference between the aspirin consumers and non-consumers (p>0.05). These data suggest that long-term regulation of the COX pathway with the common over-the-counter drug aspirin may impact aging muscle mass in a sex and muscle group specific fashion, which is supported by previous mechanistic human and animal studies.

